# Examining the role of unmeasured confounding in mediation analysis with genetic and genomic applications

**DOI:** 10.1186/s12859-017-1749-y

**Published:** 2017-07-19

**Authors:** Sharon M. Lutz, Annie Thwing, Sarah Schmiege, Miranda Kroehl, Christopher D. Baker, Anne P. Starling, John E. Hokanson, Debashis Ghosh

**Affiliations:** 1 Department of Biostatistics and Informatics, University of Colorado Anschutz Medical Campus, 13001 E. 17th Place, B119 Bldg. 500 W3128, Aurora, CO 80045 USA; 2 Department of Pediatrics and Pulmonary Medicine, Children’s Hospital Colorado, Aurora, CO USA; 3 Department of Epidemiology, University of Colorado Anschutz Medical Campus, Aurora, CO USA

**Keywords:** Mediation analysis, Mediated effects, Direct effects, Unmeasured confounding, Population stratification

## Abstract

**Background:**

In mediation analysis if unmeasured confounding is present, the estimates for the direct and mediated effects may be over or under estimated. Most methods for the sensitivity analysis of unmeasured confounding in mediation have focused on the mediator-outcome relationship.

**Results:**

The Umediation R package enables the user to simulate unmeasured confounding of the exposure-mediator, exposure-outcome, and mediator-outcome relationships in order to see how the results of the mediation analysis would change in the presence of unmeasured confounding. We apply the Umediation package to the Genetic Epidemiology of Chronic Obstructive Pulmonary Disease (COPDGene) study to examine the role of unmeasured confounding due to population stratification on the effect of a single nucleotide polymorphism (SNP) in the CHRNA5/3/B4 locus on pulmonary function decline as mediated by cigarette smoking.

**Conclusions:**

Umediation is a flexible R package that examines the role of unmeasured confounding in mediation analysis allowing for normally distributed or Bernoulli distributed exposures, outcomes, mediators, measured confounders, and unmeasured confounders. Umediation also accommodates multiple measured confounders, multiple unmeasured confounders, and allows for a mediator-exposure interaction on the outcome. Umediation is available as an R package at https://github.com/SharonLutz/Umediation A tutorial on how to install and use the Umediation package is available in the Additional file 1.

**Electronic supplementary material:**

The online version of this article (doi:10.1186/s12859-017-1749-y) contains supplementary material, which is available to authorized users.

## Background

With the recent availability of genome wide genetic and omics data in large population studies, researchers have an opportunity to interrogate the biological pathways by which specific genetic susceptibility is associated with adverse clinical outcomes. For example, there is a replicated genome wide association study (GWAS) signal in the *CHRNA5/3/B4* locus on chromosome 15q25.1 that is associated with decreased lung function (FEV_1_) [11] and cigarette smoking [12]. Mediation analysis decomposes this observed effect into a direct effect (i.e. the effect of the single nucleotide polymorphism (SNP) on FEV_1_ not through the mediator, cigarette smoking) and the mediated effect (i.e. the effect of the SNP on FEV_1_ through cigarette smoking).

However, the identification of direct and mediated effects relies on strong assumptions, including the assumption of no unmeasured confounding [25]. Most methods for the sensitivity analysis of unmeasured confounding in mediation have focused on the mediator-outcome relationship. These sensitivity analysis techniques for unmeasured confounding of the mediator-outcome relationship rely on: multiple modeling assumptions [6], sensitivity parameters involving counterfactual terms [21], specifying several sensitivity parameters [25], imposing no assumptions but providing large bounds of the estimates [17, 20], or imposing no assumptions and providing narrower bounds of the estimates [3]. The causal inference test (CIT) R package also assumes the exposure is completely randomized and checks the 4 association assumptions of the “Causality Equivalence Theorem” [15]. The left out variables error (L.O.V.E.) method [14] has been used to assess the bounds of correlation of a potential unmeasured confounder with the exposure, mediator, and outcome for a single mediator model [2] and multilevel mediation models [24]. However, these methods assume that the outcome and mediator are normally distributed and there is no exposure-mediator interaction on the outcome [2].

Most of these sensitivity analysis methods have focused on the role of unmeasured confounding of the mediator-outcome relationship since it is often assumed that the exposure is completely randomized such that there is no unmeasured confounding of the exposure-mediator and exposure-outcome relationships. However, for population based genetic association studies due to non-random mating, a subject’s DNA is not truly randomized. For example, when examining the effect of a SNP in the *CHRNA5/3/B4* locus on FEV_1_ as mediated by average cigarettes per day, unmeasured population stratification can be a confounder of the exposure-outcome and exposure-mediator relationships.

The Umediation R package uses simulation studies to examine the role of unmeasured confounding on the estimates for the direct and mediated effects allowing for unmeasured confounding of the exposure-outcome, exposure-mediator, and mediator-outcome relationships. This flexible R package allows for normally distributed or Bernoulli distributed exposures, outcomes, mediators, measured confounders, and unmeasured confounders. Umediation also accommodates multiple measured confounders, multiple unmeasured confounders, and allows for a mediator-exposure interaction on the outcome.

## Implementation

Umediation is available as an R package at https://github.com/SharonLutz/Umediation A full tutorial illustrating how to install and use the Umediation package is available in the Additional file 1.

### Input

**Fig. 1:**
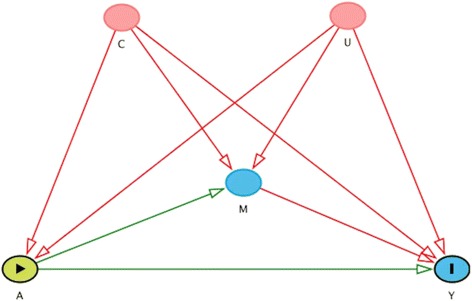
Directed Acyclic Graph showing how the data is simulated for exposure A, mediator M, outcome Y, measured confounders C, and unmeasured confounders U. This figure was generated in DAGitty [22]

The user specifies the relationship between the exposure A, the mediator M, the outcome Y, measured confounders C, and unmeasured confounders U as seen in Fig. 1. Umediation generates the continuous, normally distributed exposure A, mediator M, and outcome Y such that

$$ {E\left[ A\right]=\gamma}_0+{\gamma}_C C+{\gamma}_U U $$

$$ E\left[ M\right]={\alpha}_0+{\alpha}_A A+{\alpha}_C C+{\alpha}_U U $$

$$ E\left[ Y\right]={\beta}_0+{\beta}_A A+{\beta}_M M+{\beta}_I{A}^{\ast } M+{\beta}_C C+{\beta}_U $$

where the parameters that define the exposure A (*γ*_0_ , *γ*_*C*_ , *γ*_*U*_), the mediator M (*α*_0_ , *α*_*A*_ , *α*_*C*_ , *α*_*U*_), and the outcome Y (*β*_0_ , *β*_*A*_ , *β*_*M*_ , *β*_*I*_ , *β*_*C*_ , *β*_*U*_) are specified by the user. For dichotomous, Bernoulli distributed exposure A, mediator M, and/or outcome Y, the identity link is replaced by the logit link in the above equations. By changing the user specified values of *γ* , *α* , *β* the user is able to change the relationship between the exposure A, mediator M, outcome Y, measured confounders C, and unmeasured confounders U.

### Assumptions

For the models specifying the exposure, mediator, and outcome in the input, we are assuming that the exposure, mediator, and outcome are normally distributed or Bernoulli distributed as specified above. The user needs to make sure that these model assumptions are met and may need to transform the variables accordingly. As a result, these linear regression and logistic regression models are based on the standard assumptions regarding asymptotics and the required sample size for these models should be large enough to meet these assumptions (i.e. sample size >30).

### Output

Once this relationship is specified, the user is able to examine how the results of the mediation analysis [23] would change if the unmeasured confounder U was included or excluded from the model via simulation studies. The function outputs the proportion of simulations where the mediated or direct effect are significant when the model does not include U versus includes U, as well as the proportion of simulations where the conclusions based on the estimates match whether U is included or excluded. The function also outputs the average estimate of the mediated effect and direct effect when the model does not include U versus includes U and the average absolute difference for the estimates when U is included or excluded from the model. The correlation between all model variables is also given in order to show how the changes in *γ* , *α* , *β* effects the relationship between these variables.

### Data analysis example

**Fig. 2:**
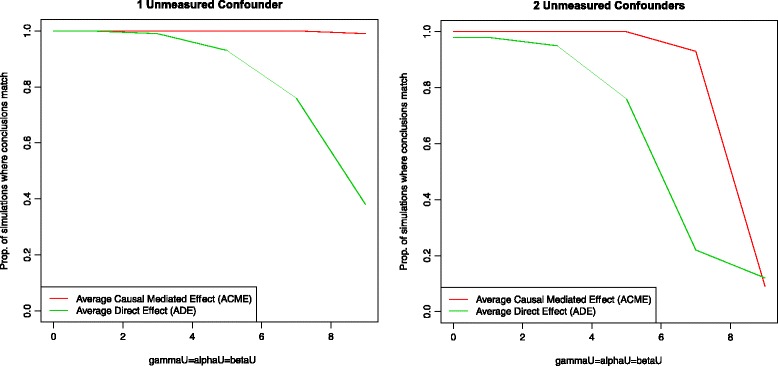
Using Umediation for one unmeasured confounder due to population stratification, the proportion of simulations where the results match for the mediated effect whether the unmeasured confounder U is included or excluded from the analysis is greater than 98% and the proportion of simulations where the results match for the direct effect is greater than 89% for an effect of confounding less than or equal to that of the observed second PC for genetic ancestry (i.e. *γ*_*U*_ = *α*_*U*_ = *β*_*U*_ ≤ 5 and U is normally distributed with mean 0 and variance 0.001). For a very strong effect (i.e. *γ*_*U*_ = *α*_*U*_ = *β*_*U*_ > 5 and both U1 and U2 are normally distributed with mean 0 and variance 0.001), then the unmeasured confounder changes the results of the mediation analysis significantly (i.e. the proportion of simulations where the results match for the direct effect whether the unmeasured confounder U is included or excluded from the model decreases to 39%). Therefore, the results of the mediation analysis would not change dramatically due to unmeasured confounding of population stratification as long as the unmeasured PC has an effect similar or less than the second measured PC of genetic ancestry. This becomes more extreme for 2 unmeasured confounders as seen in the right hand plot

In the COPDGene study, the effect of rs16969968 [chromosome 15q25.1] on FEV_1_ is mediated by average cigarettes smoked per day adjusting for known confounders: age, gender, and genetic ancestry via the first five principal components (PCs) [19]. It is possible that there is unmeasured confounding due to population stratification that is not accounted for in the first five PCs. In particular, the adjusted R squared for the mediator, average cigarettes per day, as a function of the SNP, age, gender and PCs 1–5 is 0.04 and the adjusted R squared for the outcome FEV_1_ as a function of the SNP, average cigarettes per day, age, gender, and PCs 1–5 and the exposure-mediator interaction is 0.32. In order to examine how this unmeasured confounding would affect the results of the mediation analysis, we used the Umediation package assuming one to two unmeasured PCs of genetic ancestry. As seen in Fig. 2, the results of the mediation analysis would not change dramatically due to unmeasured confounding of population stratification as long as the unmeasured PC has an effect similar or less than the second strongest measured PC of genetic ancestry. A full tutorial on how to install and use Umediation to recreate this data analysis and Fig. 2 is given in the supplement.

## Discussion

While the assumption of no unmeasured confounding is required for mediation analysis, in reality this assumption may often be violated in large population based genetic studies. Thus, it is critical to assess the likely degree of bias introduced by unmeasured confounders within any given study. With the Umediation package, users may examine the hypothetical influence of an unobserved confounding variable on the estimation of direct and mediated effects, in the presence or absence of a statistical interaction between the exposure and mediator variables. Importantly, the package is flexible enough to allow for confounders of the exposure-mediator, mediator-outcome, and exposure-outcome relationships. While the data analysis example focused on population stratification which can confound the SNP-mediator and SNP-outcome associations, the strongest potential for unmeasured confounding is lifestyle and socioeconomic factors of the mediator-outcome association, such as physical activity and education. A strength of the Umediation package is that one can simultaneously account for all of these unmeasured confounders.

### Further examples

While the data analysis example focused on confounding bias due to unobserved population stratification in the decomposition of the total effect of a SNP on lung function (FEV_1_) through the mediator cigarettes smoking, the Umediation package is applicable and useful in any scenario where there may be unmeasured confounding of the exposure-mediator-outcome relationship (i.e. the exposure was not completely randomized). For example, Umediation has many practical and clinical uses. A relevant clinical example is the pathway by which maternal preeclampsia contributes to the risk of chronic lung disease in the newborn [5]. The effects of preeclampsia (exposure A) on the developing lung (outcome Y) are mediated by disruptions in angiogenesis (mediator M), indicated by altered pro-angiogenic umbilical cord blood biomarkers [4, 13]. However, preterm birth (measured confounder C) may confound the mediator-outcome relationship, as the degree of prematurity is associated with both severe lung disease [9] and levels of pro-angiogenic biomarkers [1]. Genetic variation affects the risk of maternal preeclampsia (exposure A), angiogenesis in the infant (mediator M), and the risk for preterm lung disease (outcome Y) [10]. However, specific genetic confounders are often unmeasured (i.e. unmeasured confounders U). While we cannot measure the confounding caused by genetic variation, we can use Umediation to explore whether this confounding is largely responsible for the observed associations. That is, previously published studies may suggest minimum and maximum boundaries of the hypothesized confounder-exposure, confounder-mediator, and confounder-outcome associations. Entering these parameters into Umediation can provide adjusted estimates.

In addition, epigenetic processes have been proposed as plausible mediators linking exposures in one period of life (for example, prenatally) to health outcomes at a later stage of life (such as childhood or adulthood). For example, maternal smoking in pregnancy has been consistently associated with a greater risk of childhood overweight and obesity in the offspring [16]. Maternal smoking is also associated with numerous detectable changes in DNA methylation in umbilical cord blood at birth [8]. An investigator may therefore ask how much of the effect of maternal smoking during pregnancy (exposure A) on offspring overweight and obesity (outcome Y) is mediated through changes in DNA methylation detectable in cord blood at birth (mediator M). There may be a number of measured confounders C influencing the probability of maternal smoking and the probability of offspring obesity, such as maternal age at delivery or household income. It is also likely that there are unmeasured confounders U of the associations between maternal smoking and cord blood DNA methylation, between cord blood DNA methylation and offspring obesity, and between maternal smoking and offspring obesity. Genetic variability in the mother and offspring is a possible confounder of each of these associations, and we must consider whether this confounding is likely to be so severe as to challenge our conclusions. Again, we can do so by setting bounds on the probable magnitude of these associations, and by using Umediation to estimate bias-adjusted associations under each of these scenarios.

### Limitations

Umediation examines the role of unmeasured confounding via simulations studies by running mediation analysis [23] both with and without the unmeasured confounders U for the simulated data based on user input. This is not a theoretical approach but a simulation based approach to examine the role of unmeasured confounding of the exposure-mediator-outcome relationship. As a result, care needs to be given to the interpretation of the results and the number of simulations run. Increasing the number of simulations also increases the running time of the function. For computational efficiency, Umediation can be run in parallel on a cluster by using the seed parameter of the function to run each iteration of the simulation on separate nodes and then compiling the results after the simulations have run. This allows the Umediation function to be run for a large number of simulations in a reasonable amount of time.

Additionally, the Umediation package currently assumes no correlation between the measured confounders C and unmeasured confounders U. While this is a reasonable assumption for the data example where the measured and unmeasured confounders are PCs, the investigator needs to determine if this is a reasonable assumption for their particular question of interest. While the Umediation package allows for an exposure-mediator interaction on the outcome of interest, it is important to note that the Umediation package does not allow for interactions of the measured and unmeasured confounders with the exposure or mediator on the outcome of interest. Also, the Umediation package currently only accommodates one mediator of the exposure-outcome relationship.

### Methods that allow for unmeasured confounding

While we have focused on the effect of unmeasured confounding of the exposure-mediator-outcome relationship in mediation analysis, there are methods that allow for unmeasured confounding, such as instrumental variable methods. There have been instrumental variable methods proposed that can be extended to handle mediation analysis for continuous, normally distributed outcomes [7] and binary, Bernoulli distributed outcomes [18].

## Conclusions

Umediation allows investigators to make reasonable quantitative estimates of the magnitude of the effect due to unmeasured confounding of the exposure-mediator, mediator-outcome, or exposure-outcome associations. This R package accommodates multiple unmeasured confounders, which may be either Bernoulli or normally distributed. The utility of Umediation becomes apparent whenever the need arises to examine the impact of unmeasured variables in a mediation analysis: in a post-hoc setting, when data collection and analysis have already been conducted, or in the early stages of study design, when exploring the relative value accrued by collecting data on variables that may be costly or difficult to obtain. By estimating the degree of bias produced at the plausible upper and lower boundaries of the association between each unmeasured confounder and the exposure, mediator, or outcome, investigators will be able to assess whether the mediated or direct effects are likely to be over or under estimated due to unobserved confounders.

## Additional file

Additional file 1: Supplemental Tutorial for Umediation: an R Package for Examining the Role of Unmeasured Confounding in Mediation Analysis with Genetic and Genomic Applications. (PDF 612 kb)

## Abbreviations

COPD: Chronic Obstructive Pulmonary Disorder
FEV_1_: forced expiratory volume in the first second
GWAS: genome wide association study
PCs: principal components
SNP: single nucleotide polymorphism
