# Supplementary material for: Examining the role of unmeasured confounding in mediation analysis with genetic and genomic applications
Source: BMC Bioinformatics. 2017 Jul 19;18:344. doi: 10.1186/s12859-017-1749-y (PMC5517807; doi:10.1186/s12859-017-1749-y)
Supplement: Additional file 1: — Supplemental Tutorial for Umediation: an R Package for Examining the Role of Unmeasured Confounding in Mediation Analysis with Genetic and Genomic Applications. (PDF 612 kb) [file 12859_2017_1749_MOESM1_ESM.pdf]

Supplemental Tutorial for Umediation: an R Package for Examining the Role of Unmeasured Confounding in Mediation Analysis with Genetic and Genomic Applications

1.1 Data Example

The Genetic Epidemiology of Chronic Obstructive Pulmonary Disease (COPDGene) Study is a multicenter observational study designed to identify genetic factors associated with Chronic Obstructive Pulmonary Disease (COPD) and to characterize COPD-related phenotypes. In the COPDGene study among non-Hispanic whites, it has been shown that the effect of SNPs on chromosome 15q25 [*CHRNA5/3/B4*] on FEV_1 is mediated by average cigarettes per day controlling for age, gender and genetic ancestry via principal components (PCs) (Siedlinski et al, 2013) (Lutz and Hokanson, 2014). The Umediation package can be used to examine the role of unmeasured confounding of the exposure-mediator-outcome relationship via population stratification. For example, Umediation can be used to examine how the effect of rs16969968 on FEV_1 via the mediator, average cigarettes per day, changes if there was population stratification that was not accounted for in the 5 measured PCs.

1.2 Installation

First, the following packages must be installed in R: devtools, car, and mediation (Tingley et al. 2014). Use the following commands in R to install these 3 packages.

```
install.packages("devtools") #The devtools package must be installed first
install.packages("mediation") #The mediation package must be installed first
install.packages("car") #The car package must be installed first
```

Once these packages are installed, the following R commands download and load the Umediation package.

```
devtools::install_github("SharonLutz/Umediation")
library(Umediation)
?Umediation # For details on this function and how to choose input variables
```

1.3 Input for Umediation

To examine the role of unmeasured confounding due to population stratification using the Umediation function, one needs to specify if the variables are continuous and normally distributed (i.e. “C”) or dichotomous (i.e. “D”). In this example, for the exposure A (i.e. dominant SNP rs16969968), **Atype="D"**; for the continuous mediator M (i.e. average cigarettes per day), **Mtype=”C”**; for the continuous outcome Y (i.e. FEV_1), **Ytype=”C”**; for measured confounders (i.e. age, gender, PC1, PC2, PC3, PC4, PC5), **Ctype= c("C","D","C","C","C","C","C").** Assuming the unmeasured confounding is due to one PC which was not accounted for, then **Utype="C".** Note that, in order to generate the binary exposure A, age must first be standardized (i.e. age=(age-mean(age))/sd(age)) since it has a much larger mean and variance than the other variables. Taking the sample mean and variance of the measured confounders (i.e. age, gender, PC1, PC2, PC3, PC4, PC5), then the vectors for the mean (muC and muU) and variance (varC and varU) of the 7 measured confounders C and 1 unmeasured confounder U are the following:

```
#round(c(mean(age),mean(gender),mean(pc1),mean(pc2),
mean(pc3),mean(pc4),mean(pc5)),2)
# 0.00 0.48 0.00 0.00 0.00 0.00 0.00

#round(c(var(age),var(gender),var(pc1),var(pc2),var(pc3),var(
pc4),var(pc5)),3)
# 1.000 0.250 0.001 0.001 0.001 0.001 0.001
```

```
### Input for Umediation
muC=c(0,0.48,0,0,0,0,0)
varC=c(1,0.25,0.001,0.001,0.001,0.001,0.001)
muU=c(0)
varU=c(0.001)
```

Next, we need to specify the model for the exposure A (SNP rs16969968) as a function of the measured confounders C (ie. age, gender, PC1-5). Umediation generates the binary exposure A based on the logit(P(A=1))=gamma0+gammaC*C+gammaU*U where gamma0, gammaC, and gammaU are based on user input. Using the data from the COPDGene study below, we can get estimates of gamma0, gammaC, and varA below which are rounded and color coded to match the output. We will start by assuming the unmeasured PC has no effect on the SNP, exposure A, then **gammaU=0**.

```
# summary(glm(snp~age+gender+pc1+pc2+pc3+pc4+pc5,family=binomial()))
#              Estimate        Std. Error  z value     Pr(>|z|)
#(Intercept)  0.37857         0.03462     10.937      < 2e-16 ***
#age         -0.01090         0.02520     -0.433      0.66530
#gender       0.05190         0.05029     1.032       0.30213
#pc1          9.41083         2.12320     4.432       9.32e-06 ***
#pc2          2.26969         2.05746     1.103       0.26996
#pc3          6.45932         2.13908     3.020       0.00253 **
#pc4          0.63162         2.03796     0.310       0.75662
#pc5         -0.49150         2.05890     -0.239      0.81132

# var(snp)
# 0.2402632
```

### Input for Umediation
# logit(P(A=1))=gamma0+gammaC*C+gammaU*U
gamma0= **0.38**
gammaC= c(**-0.01,0.05,9.41,2.27,6.46,0.63,-0.49**)
gammaU= c(**0**)
varA= **0.24**

Then, we need to specify the model for the mediator M (average cigarettes per day (i.e. cig)) as a function of the exposure A (i.e. SNP rs16969968), measured confounders C (ie. age, gender, PC1-5). Umediation generates the continuous mediator M based on the equation E[M]=alpha0+alpha*A+alphaC*C+alphaU*U where alpha0, alphaA, alphaC, and alphaU are based on user input. Using the data from COPDGene below, we can get estimates of alpha0, alphaA, alphaC, and varM below which are color coded to match the output. Assuming the unmeasured PC has no effect on the mediator average cigarettes per day, then **alphaU=0**.

```
# summary(lm(cig~snp+age+gender+pc1+pc2+pc3+pc4+pc5))
#             Estimate         Std. Error  t value     Pr(>|t|)
#(Intercept)  2.73217         0.02445     111.749     < 2e-16 ***
#snp          0.14858         0.02718     5.467       4.74e-08 ***
#age          0.09272         0.01335     6.944       4.18e-12 ***
#gender      -0.35517         0.02666     -13.323     < 2e-16 ***
#pc1         -3.94668         1.08931     -3.623      0.000293 ***
#pc2         -1.91625         1.08649     -1.764      0.077827 .
#pc3          1.15943         1.08815     1.066       0.286685
#pc4          1.34724         1.08590     1.241       0.214772
#pc5          1.71687         1.08741     1.579       0.114413
#Multiple R-squared:  0.04069,      Adjusted R-squared:  0.03953

# var(cig)
# 1.223807
```

### Input for Umediation
# E[M]=alpha0+alphaA*A+alphaC*C+alphaU*U
alpha0= **2.73**
alphaA= **0.15**
alphaC= c(**0.09,-0.36,-3.95,-1.92,1.16,1.35,1.72**)
alphaU= c(**0**)
varM= **1.22**

Finally, we need to specify the model for outcome Y (i.e. FEV_1) as a function of the exposure A (i.e. SNP rs16969968), the mediator M (i.e. average cigarettes per day), measured confounders C (ie. age, gender, PC1-5). Allowing for an exposure-mediator interaction on the outcome, then interact=TRUE. Umediation generates the continuous outcome Y based on the equation E[Y]=beta0+betaA*A+betaM*M+ betaI*A*M +betaC*C+betaU*U where beta0, betaA, betaM, betaI, betaC, and betaU are based on user input. Using the data from the COPDGene study below, we can get estimates of beta0, betaA, betaM, betaI, betaC, and varY below which are color coded to match the output. Assuming the unmeasured PC has no effect on the FEV_1, outcome Y, then **betaU=0**.

```
#summary(lm(fev1~snp+cig+snp*cig+age+gender+pc1+pc2+pc3+pc4+pc5))
#              Estimate  Std. Error  t value     Pr(>|t|)
#(Intercept)  2.945473       0.040600    72.548      < 2e-16 ***
#snp         -0.211165       0.050556    -4.177      2.99e-05 ***
#cig         -0.128850       0.013866    -9.293      < 2e-16 ***
#age         -0.404273       0.009672    -41.799     < 2e-16 ***
#gender      -0.699396       0.019492    -35.881     < 2e-16 ***
#pc1          3.531680       0.786821    4.489       7.29e-06 ***
#pc2          5.583258       0.784265    7.119       1.20e-12 ***
#pc3          0.063296       0.785301    0.081       0.9358
#pc4         -0.493284       0.783772    -0.629      0.5291
#pc5         -1.072007       0.784834    -1.366      0.1720
#snp:cig      0.042635       0.017752    2.402       0.0163 *
#Multiple R-squared:  0.3214, Adjusted R-squared:  0.3204

# var(fev1)
# 0.9005715
```

```
### Input for Umediation
# E[Y]=beta0+betaA*A+betaM*M+ betaI*A*M+betaC*C+betaU*U
beta0= 2.95
betaA= -0.21
betaM= -0.13
betaI= 0.04
betaC= c(-0.40,-0.70,3.53,5.58,0.06,-0.49,-1.07)
betaU= c(0)
varY= 0.9
interact=TRUE
```

Additional Input for Umediation:

nSim is the number of simulations run for the function. The more simulations run, the more accurate the results, but this will make the function slower. For this example, we set nSim=1000.

nBoot is the number of Monte Carlo draws for nonparametric bootstrap or quasi-Bayesian approximation for the mediate function. For this example, we set nBoot=1000.

seed sets the seed used for the random generator. For this example, we use the default seed=1.

atreat sets the treatment group for the exposure A. For this example, we use the default atreat=1.

acontrol sets the control group for the exposure A. For this example, we use the default acontrol=0.

1.4 Running Umediation

Now, we can run the Umediation function using the above input to see how the one unmeasured PC affects the mediation analysis of the effect of the SNP (exposure A) on FEV_1 (outcome Y) via average cigarettes per day (mediator M).

```
# run the Umediation function
testM<-
Umediation(n=6656,Atype="D",Mtype="C",Ytype="C",Ctype=c("C","D","C","C","C","C","C")
,Utype=c("C"),interact=TRUE,muC=c(0,0.48,0,0,0,0,0),varC=c(1,0.25,0.001,0.001,0.001,
0.001,0.001),muU=c(0),varU=c(0.001),gamma0=0.38,gammaC=c(-
0.01,0.05,9.41,2.27,6.46,0.63,-0.49),gammaU=0,varA=0.24, alpha0=2.73,
alphaA=0.15,alphaC=c(0.09,-0.36,-3.95,-1.92,1.16,1.35,1.72), alphaU=0, varM=1.22,
beta0=2.95,betaA=-0.21,betaM=-0.13, betaI=0.04, betaC=c(-0.40,-0.70,3.53,5.58,0.06,-
0.49,-1.07),betaU=0,varY=0.9, alpha=0.05, nSim=1000,nBoot=1000, seed=1,atreat=1,
acontrol=0)

# output results
testM
```

Below is the output from Umediation. Since we generated the one unmeasured confounder U to have no effect on the exposure A (gammaU=0), the mediator M (alphaU=0), and the outcome (betaU=0), it is not surprising that the results of the mediation analysis match whether the unmeasured confounder U is included in the model or not for the average casual mediated effect (ACME) and the average direct effect (ADE).

```
$Results
                                                                                [,1]
Prop. of simulations w/ significant ACME excluding U                    1.0000000000
Prop. of simulations w/ significant ACME including U                    1.0000000000
Prop. of simulations where conclusions based on ACME match              1.0000000000
Average ACME excluding U                                               -0.0160622192
Average ACME including U                                               -0.0160844785
Average absolute difference of ACME including U minus ACME excluding U  0.0003895512
Prop. of simulations w/ significant ADE excluding U                     1.0000000000
Prop. of simulations w/ significant ADE including U                     1.0000000000
Prop. of simulations where conclusions based on ADE match               1.0000000000
Average ADE excluding U                                                -0.1060445415
Average ADE including U                                                -0.1062731185
Average absolute difference of ADE including U minus ADE excluding U    0.0026466043

$Correlations_Between_Variables
       A     M     Y    C1    C2    C3    C4    C5    C6    C7    U1
A   1.00  0.04 -0.03  0.02  0.01  0.16  0.03  0.10  0.02  0.00  0.00
M   0.04  1.00 -0.12  0.09 -0.16 -0.09 -0.08  0.04  0.05  0.03  0.01
Y  -0.03 -0.12  1.00 -0.36 -0.30  0.09  0.17 -0.01 -0.02 -0.02 -0.01
C1  0.02  0.09 -0.36  1.00  0.01  0.01 -0.01  0.00  0.00  0.00 -0.01
C2  0.01 -0.16 -0.30  0.01  1.00  0.02  0.02  0.01 -0.01  0.02 -0.01
C3  0.16 -0.09  0.09  0.01  0.02  1.00 -0.01 -0.01  0.01  0.03 -0.01
C4  0.03 -0.08  0.17 -0.01  0.02 -0.01  1.00  0.00 -0.01  0.01  0.02
C5  0.10  0.04 -0.01  0.00  0.01 -0.01  0.00  1.00  0.00  0.01 -0.01
C6  0.02  0.05 -0.02  0.00 -0.01  0.01 -0.01  0.00  1.00  0.00  0.01
C7  0.00  0.03 -0.02  0.00  0.02  0.03  0.01  0.01  0.00  1.00  0.00
U1  0.00  0.01 -0.01 -0.01 -0.01 -0.01  0.02 -0.01  0.01  0.00  1.00

$Warning
[1] "Warning: correlations are only valid if at least one of the variables is
normally distributed."
```

1.5 Running Umediation for Multiple Scenarios

Now we can examine the impact of one unmeasured PC by varying gammaU=alphaU=betaU from 1 to 9 by using the code below to loop through the Umediation function.

```
simU<-c(0,seq(1,9,by=2)) # create a vector of different effect sizes for U
resMat<-matrix(0,nrow=12,ncol=length(simU)) # create a matrix for results

#cycle through different effect sizes
for(jj in 1:length(simU)){

# run the Umediation function
testM<-
Umediation(n=6656,Atype="D",Mtype="C",Ytype="C",Ctype=c("C","D","C","C","C","C","C")
,Utype=c("C"),interact=TRUE,muC=c(0,0.48,0,0,0,0,0),varC=c(1,0.25,0.001,0.001,0.001,
0.001,0.001),muU=c(0),varU=c(0.001),gamma0=0.38,gammaC=c(-
0.01,0.05,9.41,2.27,6.46,0.63,-0.49),gammaU=c(simU[jj]), varA=0.24, alpha0=2.73,
alphaA=0.15,alphaC=c(0.09,-0.36,-3.95,-1.92,1.16,1.35,1.72), alphaU=c(simU[jj]),
varM=1.22,beta0=2.95,betaA=-0.21,betaM=-0.13, betaI=0.04, betaC=c(-0.40,-
0.70,3.53,5.58,0.06,-0.49,-1.07),betaU=c(simU[jj]),varY=0.9, alpha=0.05, nSim=100,
nBoot=100,seed=1,atreat=1,acontrol=0)

resMat[,jj]<-testM$Results
}
write.table(resMat,file="medU1",row.names=FALSE,quote=FALSE) # save results
```

Then we can examine the impact of 2 unmeasured PCs by varying gammaU=alphaU=betaU from 1 to 9 by using the code below to loop through the Umediation function.

```
simU<-c(0,seq(1,9,by=2)) # create a vector of different effect sizes for U
resMat2<-matrix(0,nrow=12,ncol=length(simU)) # create a matrix for results

#cycle through different effect sizes
for(jj in 1:length(simU)){

# run the Umediation function
testM<-
Umediation(n=6656,Atype="D",Mtype="C",Ytype="C",Ctype=c("C","D","C","C","C","C","C")
,Utype=c("C","C"),interact=TRUE,muC=c(0,0.48,0,0,0,0,0),varC=c(1,0.25,0.001,0.001,0.
001,0.001,0.001),muU=c(0,0),varU=c(0.001,0.001),gamma0=0.38,gammaC=c(-
0.01,0.05,9.41,2.27,6.46,0.63,-0.49),gammaU=c(simU[jj],simU[jj]), varA=0.24,
alpha0=2.73,alphaA=0.15,alphaC=c(0.09,-0.36,-3.95,-1.92,1.16,1.35,1.72),
alphaU=c(simU[jj],simU[jj]),varM=1.22,beta0=2.95,betaA=-0.21,betaM=-0.13,
betaI=0.04,betaC=c(-0.40,-0.70,3.53,5.58,0.06,-0.49,-1.07),
betaU=c(simU[jj],simU[jj]),varY=0.9,alpha=0.05,nSim=100,nBoot=100,seed=1,atreat=1,ac
ontrol=0)

resMat2[,jj]<-testM$Results
}
write.table(resMat2,file="medU2",row.names=FALSE,quote=FALSE) # save results
```

1.6a Plots for 1 Unmeasured Confounder

Below are the commands to create the following plots in R for one unmeasured confounder to show the proportion of simulations where the results match and the average difference in estimates.

```
ress<-read.table("medU1",header=TRUE) # read in saved results
rownames(ress)<-c("Prop. of simulations w/ significant ACME excluding U","Prop. of
simulations w/ significant ACME including U","Prop. of simulations where conclusions
based on ACME match","Average ACME excluding U","Average ACME including U","Average
absolute difference of ACME including U minus ACME excluding U","Prop. of
simulations w/ significant ADE excluding U","Prop. of simulations w/ significant ADE
including U","Prop. of simulations where conclusions based on ADE match","Average
ADE excluding U","Average ADE including U","Average absolute difference of ADE
including U minus ADE excluding U")
ress # output results
simU<-c(0,seq(1,9,by=2))

# create plot where proportion of plots match in working/home directory
pdf("matchU1.pdf")
plot(simU,ress[3,],lty=1,type="l",ylim=c(0,1),main="1 Unmeasured
Confounder",ylab="Prop. of simulations where conclusions
match",xlab=c("gammaU=alphaU=betaU"),col=2)
lines(simU,ress[9,],lty=1,type="l",col=3)
legend("bottomleft",c("Average Causal Mediated Effect (ACME)","Average Direct Effect
(ADE)"),lty=1,col=c(2,3))
dev.off()

# create plot for the average difference in effects in working/home directory
pdf("DiffEffectU1.pdf")
plot(simU,ress[6,],lty=1,type="l",ylim=c(0,0.12),main="1 Unmeasured
Confounder",ylab="Average absolute difference in
effect",xlab=c("gammaU=alphaU=betaU"),col=2)
lines(simU,ress[12,],lty=1,type="l",col=3)
legend("topleft",c("Average Causal Mediated Effect (ACME)","Average Direct Effect
(ADE)"),lty=1,col=c(2,3))
dev.off()
```

Below are the plots created by these commands for one unmeasured confounder.

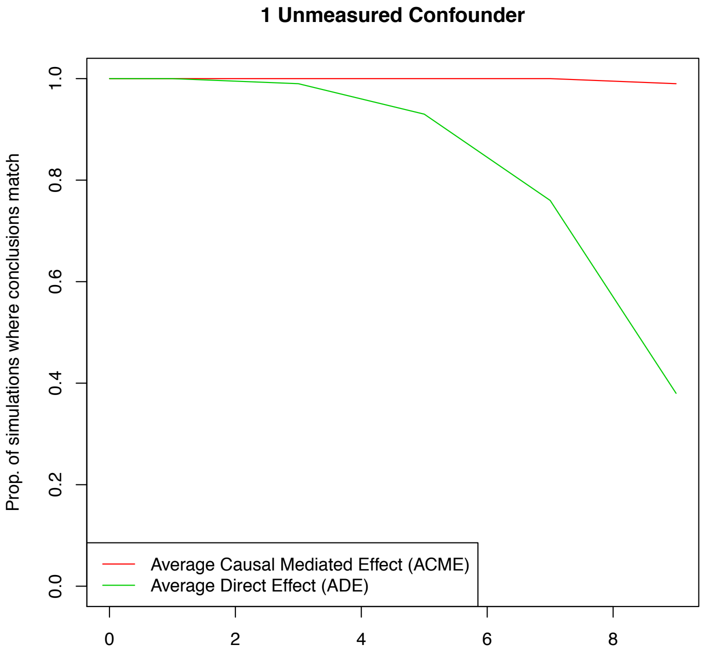

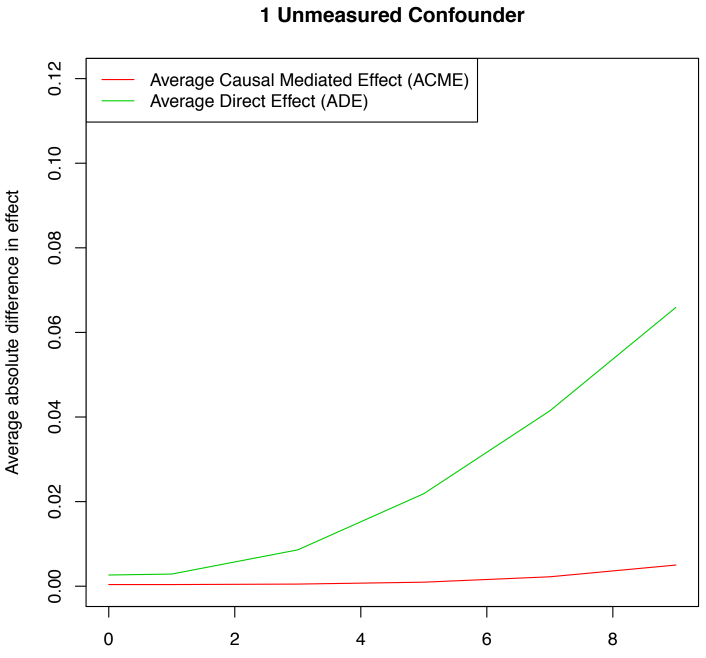

1.6b Plots for 2 Unmeasured Confounders

Below are the commands to create the following plots in R for 2 unmeasured confounders to show the proportion of simulations where the results match and the average difference in estimates.

```
ress<-read.table("medU2",header=TRUE) # read in saved results
rownames(ress)<-c("Prop. of simulations w/ significant ACME excluding U","Prop. of simulations w/ significant ACME including U","Prop. of simulations where conclusions based on ACME match","Average ACME excluding U","Average ACME including U","Average absolute difference of ACME including U minus ACME excluding U","Prop. of simulations w/ significant ADE excluding U","Prop. of simulations w/ significant ADE including U","Prop. of simulations where conclusions based on ADE match","Average ADE excluding U","Average ADE including U","Average absolute difference of ADE including U minus ADE excluding U")
ress # output results

simU<-c(0,seq(1,9,by=2))

# create plot where proportion of plots match in working/home directory
pdf("matchU2.pdf")
plot(simU,ress[3,],lty=1,type="l",ylim=c(0,1),main="2 Unmeasured Confounders",ylab="Prop. of simulations where conclusions match",xlab=c("gammaU=alphaU=betaU"),col=2)
lines(simU,ress[9,],lty=1,type="l",col=3)
legend("bottomleft",c("Average Causal Mediated Effect (ACME)","Average Direct Effect (ADE)"),lty=1,col=c(2,3))
dev.off()

# create plot for the average difference in effects in working/home directory
pdf("DiffEffectU2.pdf")
plot(simU,ress[6,],lty=1,type="l",ylim=c(0,0.12),main="2 Unmeasured Confounders",ylab="Average absolute difference in effect",xlab=c("gammaU=alphaU=betaU"),col=2)
lines(simU,ress[12,],lty=1,type="l",col=3)
legend("topleft",c("Average Causal Mediated Effect (ACME)","Average Direct Effect (ADE)"),lty=1,col=c(2,3))
dev.off()
```

Below are the plots created by these commands for 2 unmeasured confounders.

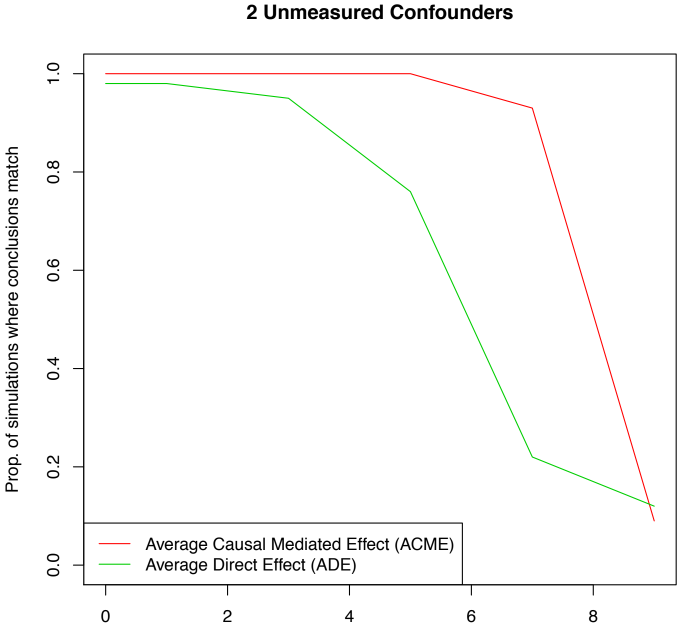

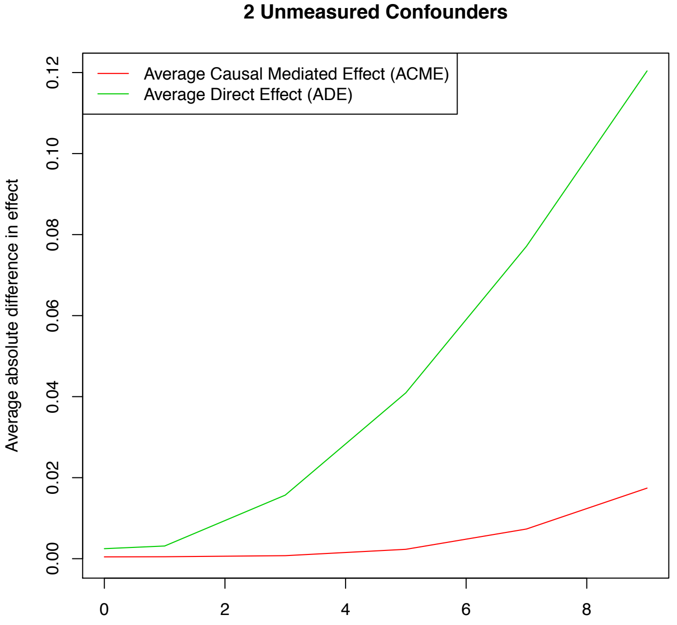

1.7 Interpreting the Results

Based on this analysis, we can see that unmeasured confounding due to population stratification does not change the results of the mediation analysis significantly unless the unmeasured confounder U has a large effect size. As seen in the plot below (row 1, column 1) for one unmeasured confounder due to population stratification, the proportion of simulations where the results match for the mediated effect whether the unmeasured confounder U is included or excluded from the analysis is greater than 98% and the proportion of simulations where the results match for the direct effect is greater than 89% for an effect of confounding less than or equal to that of the observed second PC for genetic ancestry (i.e. gammaU=alphaU=betaU less than or equal to 5). For a very strong effect (i.e. gammaU=alphaU=betaU greater than 5), then the unmeasured confounder changes the results of the mediation analysis significantly (i.e. the proportion of simulations where the results match for the direct effect whether the unmeasured confounder U is included or excluded from the model decreases to 39%). As seen in the plot below (row 2, column 1), the absolute difference in the mediated effect does not differ substantially whether the unmeasured confounder is included or excluded from the model. However, the absolute difference of the direct effect whether the unmeasured confounder is included or excluded from the model increases steadily as the effect of the unmeasured confounder increases. Therefore, the results of the mediation analysis would not change dramatically due to unmeasured confounding of 1 PC as long as that PC has an effect similar or less than the second measured PC of genetic ancestry. If the unmeasured confounder has an effect similar to the first measured PC, then the results of the mediation analysis will significantly differ by not accounting for this unmeasured confounding due to population stratification. This becomes more extreme for 2 unmeasured confounders as seen in the plots below (row 1, column 2 & row 2, column 2).

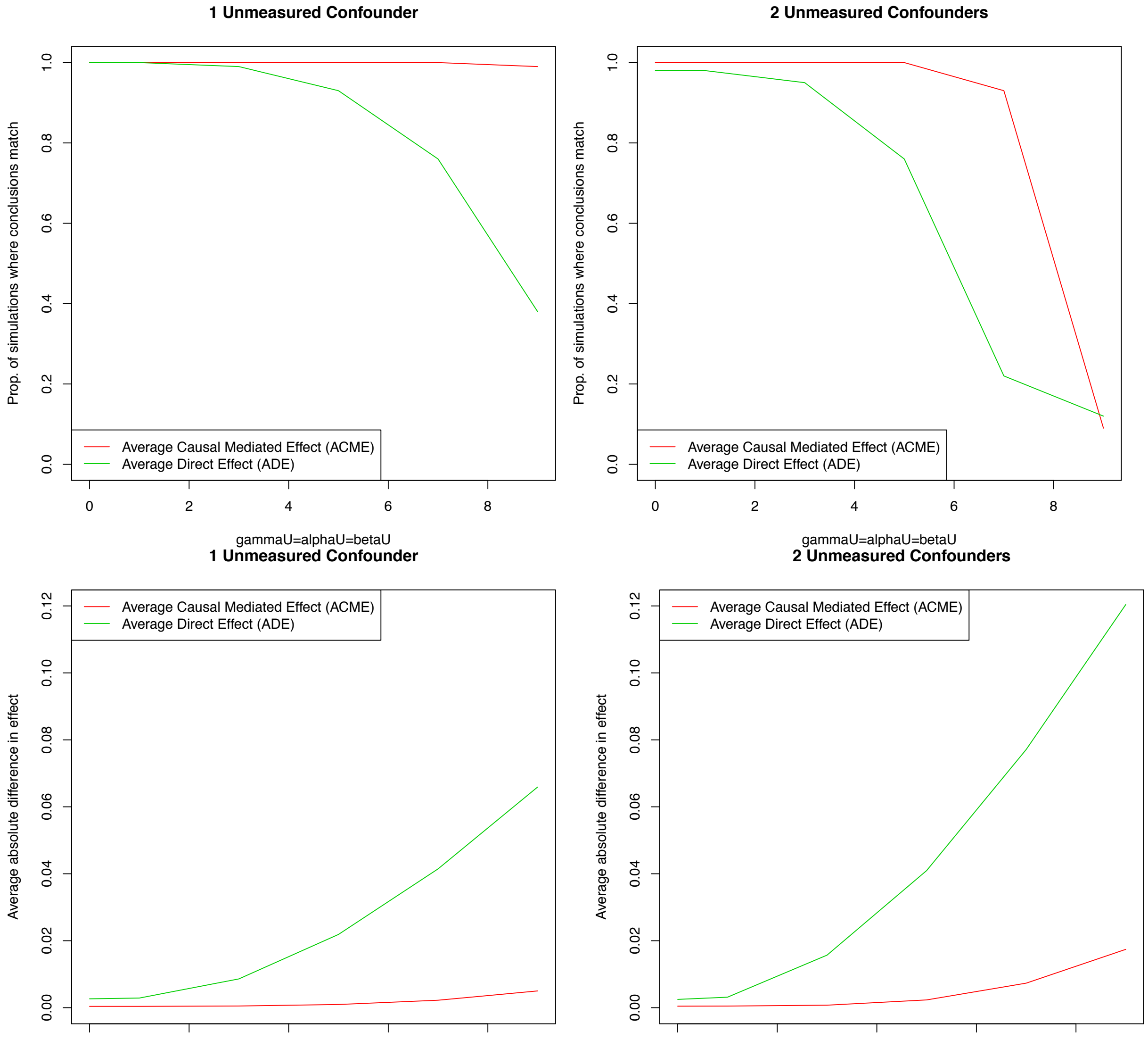

References

1. Lutz, S., Hokanson, J. (2014) Genetic Influences on Smoking and Clinical Disease. Understanding Behavioral and Biological Pathways with Mediation Analysis. Annals of the American Thoracic Society, 11(7), pp. 1082–1083.

2. Siedlinski, M. et al. (2013) Dissecting direct and indirect genetic effects on chronic obstructive pulmonary disease (COPD) susceptibility. Human Genetics, 4 (132), 431-441.

3. Tingley, D. et al. (2014). Mediation: R package for causal mediation analysis. Journal of Statistical Software, 59, 1-38.
